# New Preclinical Antimalarial Drugs Potently Inhibit Hepatitis C Virus Genotype 1b RNA Replication

**DOI:** 10.1371/journal.pone.0072519

**Published:** 2013-08-30

**Authors:** Youki Ueda, Midori Takeda, Kyoko Mori, Hiromichi Dansako, Takaji Wakita, Hye-Sook Kim, Akira Sato, Yusuke Wataya, Masanori Ikeda, Nobuyuki Kato

**Affiliations:** 1 Department of Tumor Virology, Okayama University Graduate School of Medicine, Dentistry, and Pharmaceutical Sciences, Shikata-cho, Okayama, Japan; 2 Department of Virology II, National Institute of Infectious Disease, Toyama, Shinjuku-ku, Tokyo, Japan; 3 Department of Drug Informatics, Faculty of Pharmaceutical Sciences, Okayama University, Tsushima-naka, Okayama, Japan; Kobe University, Japan

## Abstract

**Background:**

Persistent hepatitis C virus (HCV) infection causes chronic liver diseases and is a global health problem. Although new triple therapy (pegylated-interferon, ribavirin, and telaprevir/boceprevir) has recently been started and is expected to achieve a sustained virologic response of more than 70% in HCV genotype 1 patients, there are several problems to be resolved, including skin rash/ageusia and advanced anemia. Thus a new type of anti-HCV drug is still needed.

**Methodology/Principal Findings:**

Recently developed HCV drug assay systems using HCV-RNA-replicating cells (e.g., HuH-7-derived OR6 and Li23-derived ORL8) were used to evaluate the anti-HCV activity of drug candidates. During the course of the evaluation of anti-HCV candidates, we unexpectedly found that two preclinical antimalarial drugs (N-89 and its derivative N-251) showed potent anti-HCV activities at tens of nanomolar concentrations irrespective of the cell lines and HCV strains of genotype 1b. We confirmed that replication of authentic HCV-RNA was inhibited by these drugs. Interestingly, however, this anti-HCV activity did not work for JFH-1 strain of genotype 2a. We demonstrated that HCV-RNA-replicating cells were cured by treatment with only N-89. A comparative time course assay using N-89 and interferon-α demonstrated that N-89-treated ORL8 cells had more rapid anti-HCV kinetics than did interferon-α-treated cells. This anti-HCV activity was largely canceled by vitamin E. In combination with interferon-α and/or ribavirin, N-89 or N-251 exhibited a synergistic inhibitory effect.

**Conclusions/Significance:**

We found that the preclinical antimalarial drugs N-89 and N-251 exhibited very fast and potent anti-HCV activities using cell-based HCV-RNA-replication assay systems. N-89 and N-251 may be useful as a new type of anti-HCV reagents when used singly or in combination with interferon and/or ribavirin.

## Introduction

Hepatitis C virus (HCV) infection causes chronic hepatitis, which can lead to liver cirrhosis and hepatocellular carcinoma. Approximately 170 million people are infected with HCV worldwide, making HCV infection a serious global health problem [1]. HCV is an enveloped virus with a positive single-stranded RNA genome, and belongs to the *Flaviviridae* family. The HCV genome encodes a large polyprotein precursor of approximately 3000 amino acids, which is cleaved into 10 proteins in the following order: Core, envelope 1 (E1), E2, p7, non-structural 2 (NS2), NS3, NS4A, NS4B, NS5A, and NS5B [2], [3].

Until last year, the combination of pegylated-interferon (PEG-IFN) with ribavirin (RBV) was the standard therapy, resulting in a sustained virologic response (SVR) in about half of the patients receiving this treatment [4]. Two inhibitors of HCV NS3-4A protease, telaprevir and boceprevir, were recently approved as the first directly acting antiviral reagents for the treatment of HCV genotype 1, and have been used in combination with PEG-IFN and RBV [5]. The SVR rate in the treatment of HCV genotype 1 using the new triple therapy is expected to be more than 70% [6], [7]. However, several severe side effects have appeared, such as skin rash by telaprevir, ageusia by boceprevir, and advanced anemia by telaprevir/boceprevir [6], [7]. Furthermore, the rapid emergence of resistant viruses by treatment with telaprevir or boceprevir is also a serious problem [8], [9], since it is expected that these resistant viruses will exhibit a resistant phenotype against other NS3-4A inhibitors developed in the future [10]. Therefore, a new type of anti-HCV reagent without severe side effects or emergence of resistant virus is still needed [10], although several anti-HCV candidates, such as NS5A and NS5B inhibitors, are currently in phase II–III development [11].

To date, human hepatoma cell line HuH-7-derived cells are used as the only the preferred culture system for robust HCV replication, and most studies on anti-HCV reagents are currently carried out using an HuH-7-derived cell culture system [12]. We also developed an HuH-7-derived drug assay system (OR6), in which genome-length HCV-RNA (O strain of genotype 1b derived from an HCV-positive healthy carrier) encoding renilla luciferase (RL) efficiently replicates [13]. Such reporter assay systems could save time and facilitate the mass screening of anti-HCV reagents, since the values of luciferase correlated well with the level of HCV RNA after treatment with anti-HCV reagents [13]. Furthermore, OR6 assay system became more useful as a drug assay system [14] than the HCV subgenomic replicon-based reporter assay systems developed to date [12], [15], because the older systems lack the core-NS2 regions containing structural proteins likely to be involved in the events that take place in the HCV-infected human liver. Indeed, by the screening of preexisting drugs using the OR6 assay system, we have identified mizoribine [16], statins [17], hydroxyurea [18], and teprenone [19] as new anti-HCV drug candidates, indicating that the OR6 assay system is useful for the discovery of anti-HCV reagents.

On the other hand, we recently found a new human hepatoma cell line, Li23, that enables efficient HCV-RNA replication and persistent HCV production, and we developed Li23-derived assay systems (ORL8 and ORL11) [20] that are comparable to the OR6 assay system [13]. Since we indicated that the gene expression profile of Li23 cells was distinct from that of HuH-7 cells [21], we expected that anti-HCV targets in Li23-derived cells might be distinct from those in HuH-7-derived cells. Indeed, we recently found that 10 µM (a clinically achievable concentration) of RBV efficiently inhibited HCV-RNA replication in the ORL8/ORL11 assays, but not in the OR6 assay [22]. This finding led us to clarify the anti-HCV mechanism of RBV [22], [23]. Furthermore, we demonstrated that plural assay systems including OR6 and ORL8 were required for the objective evaluation of anti-HCV reagents [24]. In that study, we observed that the antimalarial drug artemisinin possessed weak anti-HCV activity, as reported previously [25].

From these results, we considered that antimalarial drugs might be good candidates for anti-HCV reagents, since the proliferation of both HCV and malaria generally occurs in hepatocytes. We therefore examined the anti-HCV activity of two preclinical antimalarial drugs, N-89 and its derivative water soluble N-251, which were previously discovered by our group as promising antimalarial reagents [26]–[28]. Here we report that N-89 and N-251 exhibit very fast and potent anti-HCV activities and have promise as potential anti-HCV drugs.

## Materials and Methods

### Cell Culture

RSc and D7 cells were derived from the cell lines HuH-7 and Li23, respectively, were cultured as described previously [20], [29]. HuH-7-derived OR6 [13], AH1R [30], and 1B-4R [Ikeda et al., submitted] cells harboring genome-length HCV-RNA and HuH-7-derived polyclonal sOR [31], and RSc-JRN/35B [Ikeda et al., submitted] cells harboring an HCV subgenomic replicon were cultured with medium in the presence of G418 (0.3 mg/ml; Geneticin, Invitrogen, Carlsbad, CA) as described previously [13]. Li23-derived ORL8 [20], ORL11 [20], 1B-4RL [Ikeda et al., submitted], and KAH5RL [Ikeda et al., submitted] cells harboring genome-length HCV-RNA were maintained with medium in the presence of G418 (0.3 mg/ml) as described previously [20]. Li23-derived polyclonal sORL8 and sORL11 cells harboring an HCV replicon, which were established by the transfection of ORN/3-5B/QR,KE,SR RNA into the cured OL8 and OL11 cells, respectively, were also cultured with medium in the presence of G418 (0.3 mg/ml) as described previously [20]. Cured cells, from which the HCV-RNA had been eliminated by IFN treatment, were also maintained with medium in the absence of G418 as described previously [13]. HCV-RNA-replicating cells possess the G418-resistant phenotype because neomycin phosphotransferase as a selective marker was produced by the efficient replication of HCV-RNA. Therefore, when HCV-RNA is excluded from the cells or when its level is decreased, the cells are killed in the presence of G418.

### Reagents

N-89 and N-251 were synthesized according to the methods described previously [26]–[28]. RBV was kindly provided by Yamasa (Chiba, Japan). Human IFN-α and vitamin E (VE) were purchased from Sigma-Aldrich (St. Louis, MO). Cyclosporine A (CsA) was purchased from Tokyo Chemical Industry (Tokyo, Japan). Artemisinin was purchased from Alexis Biochemicals (San Diego, CA).

**Figure 5:**
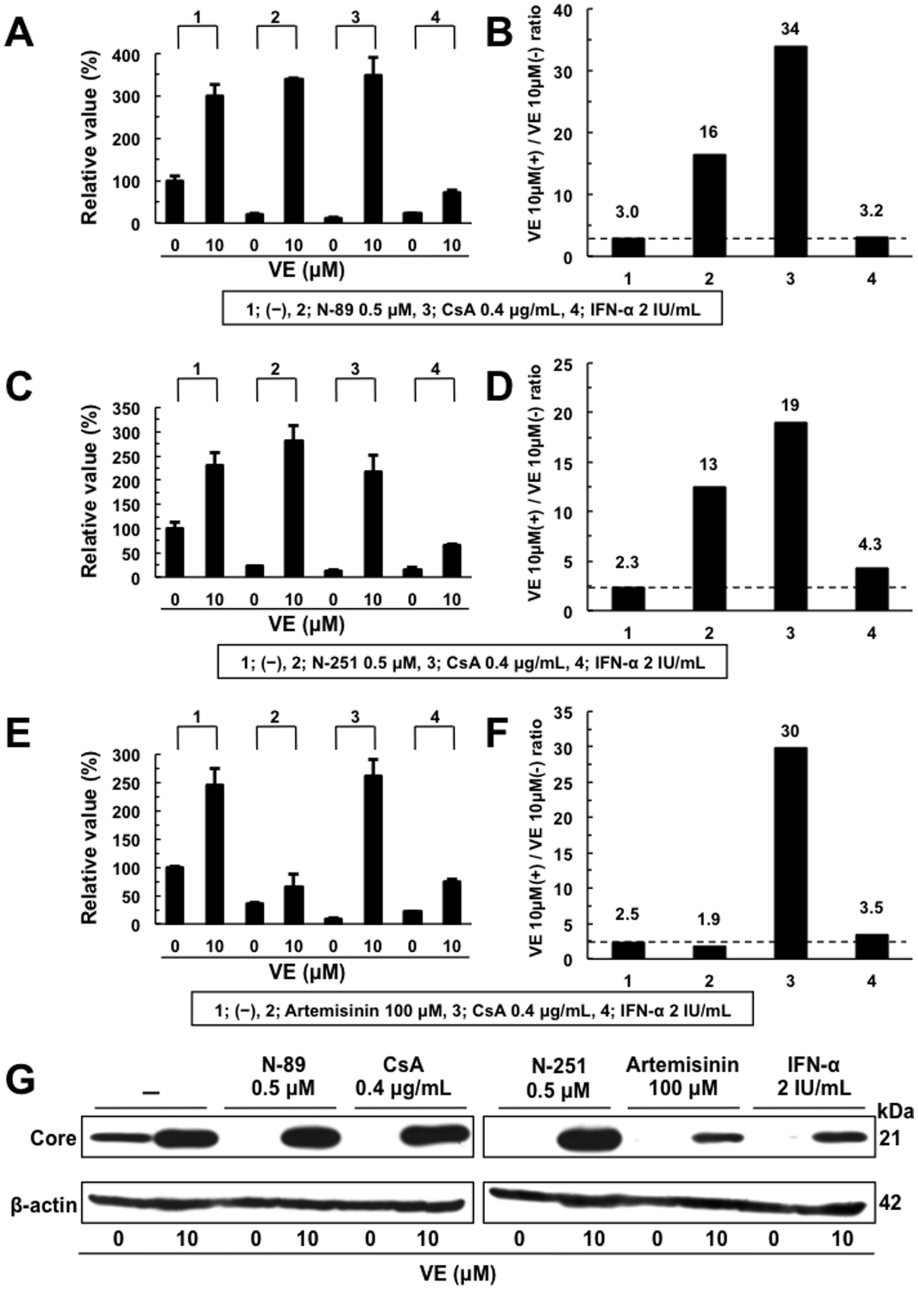
The anti-HCV activity of N-89 or N-251 was canceled by addition of VE. Effect of VE on the anti-HCV activity of N-89 (A), N-251 (C), Artemisinin (E), CsA, or IFN-α at the expected EC_90_. ORL8 cells were treated with control medium (−), N-89, CsA, or IFN-α in either the absence or presence of VE for 72 hrs. After treatment, an RL assay of harvested ORL8 cell samples was performed. (B, D, and F) The ratio of RL activity in the presence of VE to the RL activity in the absence of VE. The above ratio was calculated from the data of (A, C, and E). The horizontal line indicates the promoting effect of VE alone on HCV-RNA replication as a baseline. (G) Western blot analysis was performed as described in Fig. 1B.

### RL Assay

RL assay was performed as described previously [20], [24]. Briefly, the cells were plated onto 24-well plates (2×10^4^ cells per well) in triplicate and then treated with each reagent at several concentrations for 72 hrs. After treatment, the cells were subjected to luciferase assay using the RL assay system (Promega, Madison, WI). The experiments were performed at least in triplicate. From the assay results, the 50% effective concentration (EC_50_) of each reagent was determined.

### WST-1 Cell Proliferation Assay

The WST-1 cell proliferation assay was performed as described previously [24]. Briefly, The cells were plated onto 96-well plates (1×10^3^ cells per well) in triplicate and then treated with each reagent at several concentrations for 72 hrs. After treatment, the cells were subjected to the WST-1 cell proliferation assay (Takara Bio, Otsu, Japan) according to the manufacturer’s protocol. This assay is based on the enzymatic cleavage of the tetrazolium salt WST-1 to formazan by cellular mitochondrial dehydrogenases present in viable cells. Therefore, there are viable cells even if the value of the WST-1 assay becomes zero. The experiments were performed at least in triplicate. From the assay results, the 50% cytotoxic concentration (CC_50_) of each reagent was determined.

### Western Blot Analysis

The preparation of cell lysates, sodium dodecyl sulfate-polyacrylamide gel electrophoresis, and immunoblotting analysis were performed as previously described [32]. The antibodies used in this study were those against HCV Core (CP11; Institute of Immunology, Tokyo, Japan), NS5B (a generous gift from Dr. M. Kohara, Tokyo Metropolitan Institute of Medical Science), and β-actin (AC-15; Sigma-Aldrich) as the control for the amount of protein loaded per lane.

### Selective Index (SI)

The SI value of each reagent was determined by dividing the CC_50_ value by the EC_50_ value.

### Quantitative RT-PCR Analysis

The RNAs from HCV-RNA replicating cell lines were prepared with an RNeasy extraction kit (Qiagen). The quantitative RT-PCR analysis for HCV-RNA was performed using a real-time LightCycler PCR (Roche Diagnostics, Basel, Switzerland) as described previously [13], [20].

### HCV Infection

HCV infection was performed as described previously [29]. RSc and D7 cells were inoculated with supernatant from RSc cells replicating JR/C5B/BX-2 [29].

### Statistical Analysis

Determination of the significance of differences among groups was assessed using the Student’s *t*-test. *P*<0.05 was considered significant.

## Results

### Preclinical Antimalarial Drugs, N-89 and N-251, Showed Potent Anti-HCV Activities in Both HuH-7- and Li23-derived Genome-length HCV-RNA-replicating Cells

**Figure 1:**
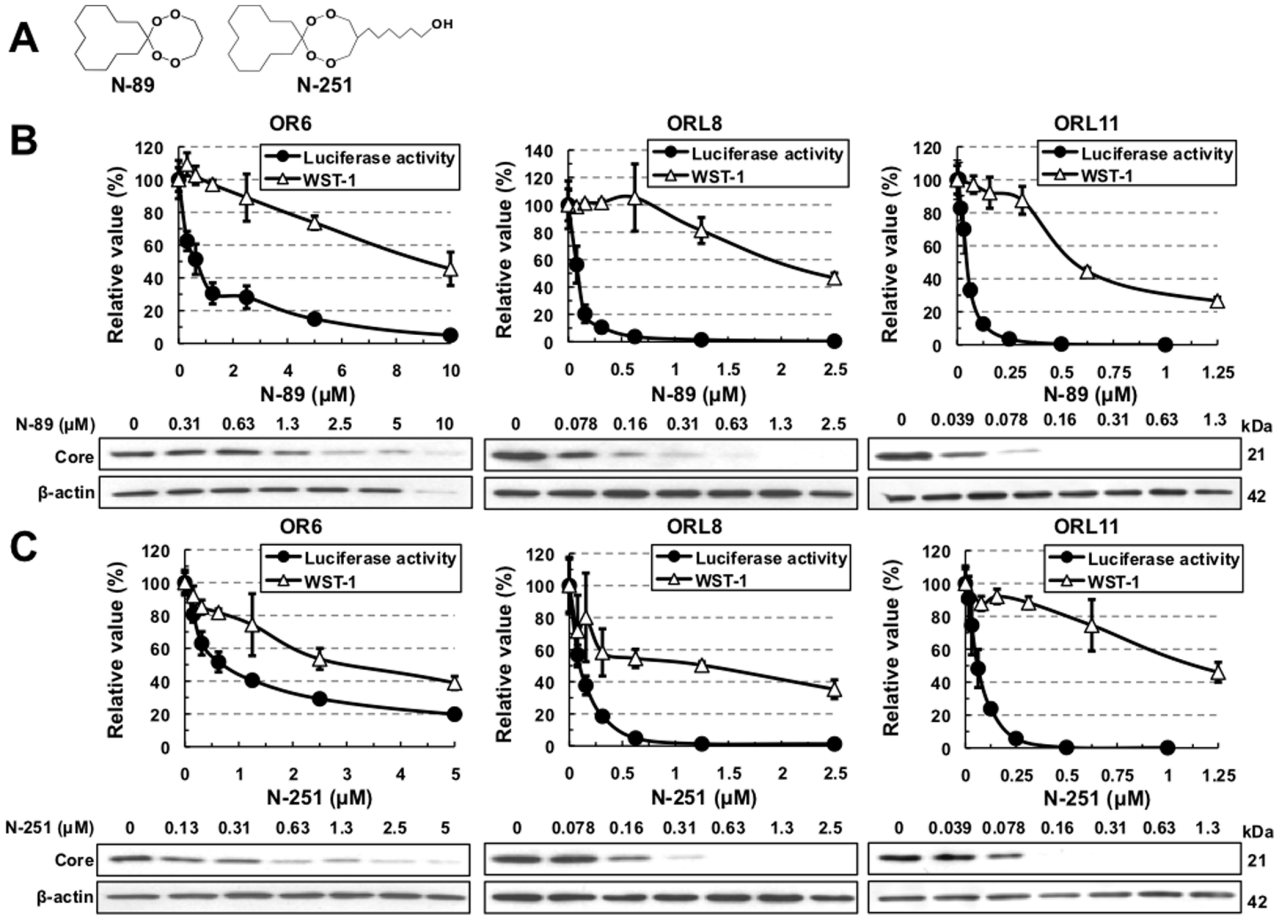
Anti-HCV activities of N-89 and N-251 detected in the OR6, ORL8, and ORL11 assays. (A) Structures of N-89 and N-251. (B) Effects of N-89 on genome-length HCV-RNA replication. OR6, ORL8, and ORL11 cells were treated with N-89 for 72 hrs, followed by RL assay (black circles in the upper panel) and WST-1 assay (open triangles in the upper panel). The relative value (%) calculated at each point, when the level in non-treated cells was assigned as 100%, is presented here. Data are expressed as the means±standard deviation of triplicate assays. Western blot analysis of the treated cells for the HCV Core was also performed (lower panel). β-actin was used as a control for the amount of protein loaded per lane. (C) Effects of N-251 on genome-length HCV-RNA replication. The RL assay, WST-1 assay, and Western blot analysis were performed as described in (B).

Recently we demonstrated that plural HCV assay systems developed using both HuH-7 and Li23 cell lines or HCV strains belonging to genotype 1b are required for the objective evaluation of anti-HCV candidates [24]. In the present work, we used our previously developed HCV assay systems to evaluate preclinical antimalarial drugs (N-89 and N-251). N-89 (1,2,6,7-Tetraoxaspiro[7.11]nonadecane) is a chemically synthesized endoperoxide compound (Fig. 1A) with potent antimalarial activity against *Plasmodium falciparum in vitro* and *Plasmodium berghei in vivo*, and it shows low levels of cytotoxicity in mice and rats (50% lethal dose: >2000 mg/kg) [26], [33], [34]. N-251 (6-(1,2,6,7-tetraoxaspiro[7.11]nonadec-4-yl)hexan-1-ol), which bears a functional side chain hydroxyl group that allows derivatization, is synthesized by replacing the hydrogen at C-4 of N-89 with hexanol (Fig. 1A), and it is as potent as N-89 against malaria parasites [27], [28]. We first evaluated the anti-HCV activities of N-89 and N-251 using HuH-7-derived OR6 and Li23-derived ORL8 and ORL11 assay systems. The results revealed that both N-89 and N-251 possessed strong anti-HCV activities (Fig. 1B and C). The EC_50_ and SI values of N-89 in each assay were calculated (EC_50_ 0.66 µM, SI 14 in OR6 assay; EC_50_ 0.089 µM, SI 26 in ORL8 assay; EC_50_ 0.045 µM, SI 12 in ORL11 assay) (Table 1), and the anti-HCV activity of N-251 was found to be as potent as that of N-89 (Table 1). The anti-HCV activities of N-89 and N-251 were confirmed by Western blot analysis of HCV Core (Fig. 1B and C). To further evaluate the activities of N-89 and N-251, as additional assay systems, we used HuH-7-derived 1B-4R (1B-4 strain [31] of genotype 1b derived from an HCV-positive healthy carrier) [Ikeda et al., submitted] and AH1R (an AH1 strain [35] of genotype 1b derived from a patient with acute hepatitis C) [30], and Li23-derived 1B-4RL (1B-4 strain [31]) and KAH5RL (KAH5 strain [31] of genotype 1b derived from a patient with acute hepatitis C) [Ikeda et al., submitted]. These assays also showed that N-89 and N-251 possessed potent anti-HCV activities (Fig. S1A–D and Table 1). It was noteworthy that N-89 exhibited the strongest anti-HCV activity (EC_50_ 0.025 µM; SI >20) in the AH1R assay (Fig. S1A and Table 1). These results suggest that the anti-HCV activity of N-89 or N-251 is not influenced by the cell line or HCV strain. We next examined the activities of N-89 and N-251 using polyclonal cell-based assay systems (HuH-7-derived sOR [31], Li23-derived sORL8 and sORL11 [22]) that facilitate the monitoring replication of HCV subgenomic replicon RNA. These assays also showed that N-89 and N-251 possessed anti-HCV activity with EC_50_ values of less than 1 µM (Fig. S1E-G and Table 1). Taken together, these results indicate that the anti-HCV activities of N-89 and N-251 are not dependent on the specific cloned cell line or HCV structural proteins.

**Table 1:** Anti-HCV activities of N-89 or N-251 in various HCV drug assay systems.

Cell origin	HuH-7						Li23							
HCV strain	O		1B-4		AH1		O		O		1B-4		KAH5	
Assay	OR6		1B-4R		AH1R		ORL8		ORL11		1B-4RL		KAH5RL	
Reagents	9.0* ^1^	14* ^3^	9.3	22	>0.5	>20	2.3	26	0.56	12	2.4	20	2.5	13
N-89	0.66* ^2^		0.42		0.025		0.089		0.045		0.12		0.19	
N-251	3.0	4.4	3.8	3.9	0.49	3.5	1.3	13	1.1	19	1.9	8.3	2.8	10
	0.69		0.98		0.14		0.10		0.059		0.23		0.29	
HCV strain	O						O		O					
Assay	sOR						sORL8		sORL11					
Reagents	1.7	2.9					1.1	9.2	1.7	14				
N-89	0.58						0.12		0.12					
N-251	2.2	3.2					4.1	19	3.1	11				
	0.69						0.22		0.27					

* ^1^CC_50_ value (µM),

* ^2^EC_50_ value (µM),

* ^3^SI value.

### N-89 and N-251 Inhibited Authentic HCV-RNA Replication

**Figure 2:**
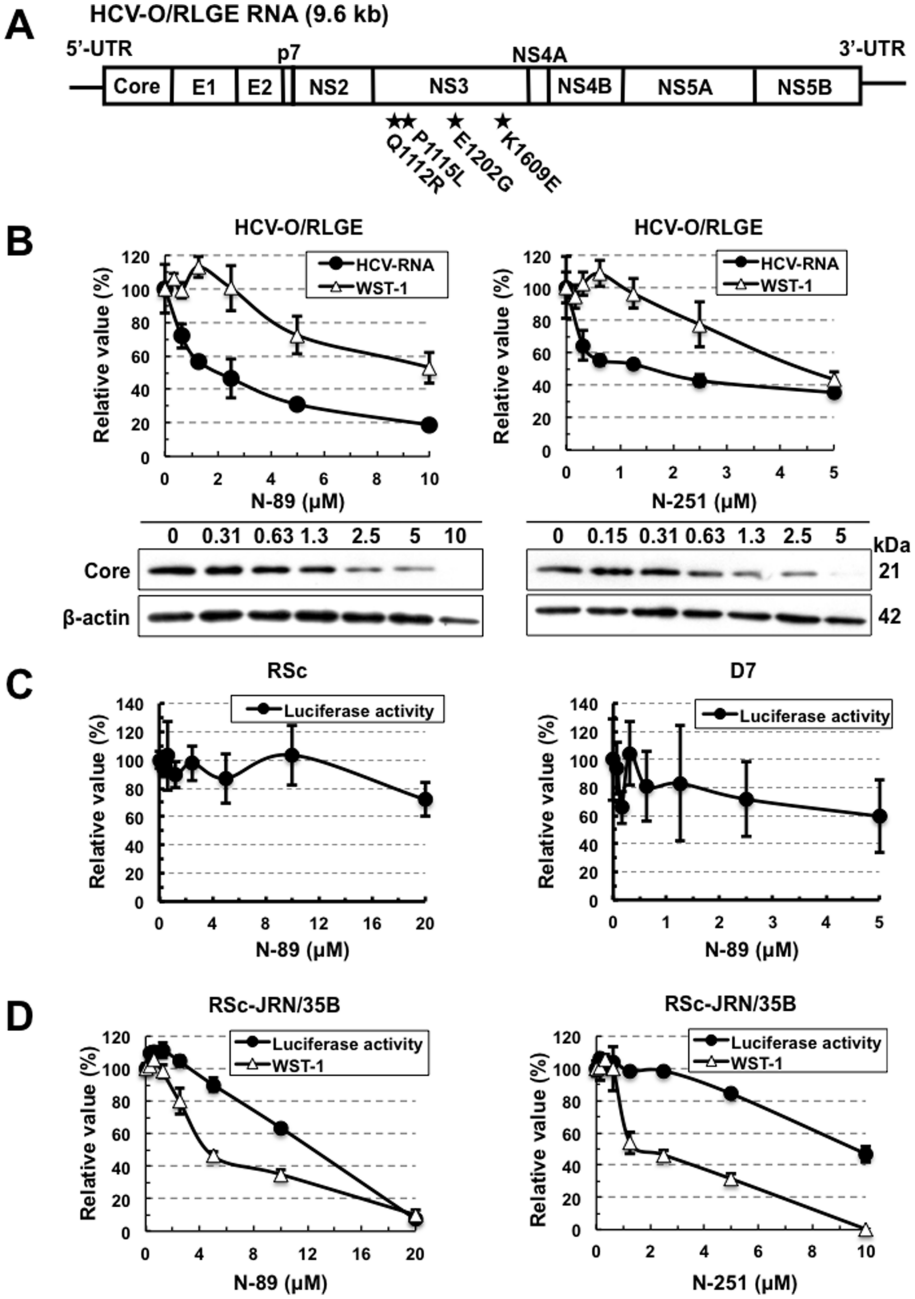
Characterization of anti-HCV activities of N-89 and N-251. (A) Schematic gene organization of authentic HCV-RNA (HCV-O/RLGE). The positions of four adaptive mutations - Q1112R, P1115L, E1202G, and K1609E - are indicated by a black star. (B) N-89 and N-251 inhibited authentic HCV-RNA replication. The cells harboring HCV-O/RLGE RNA [19] were treated with N-89 (left panel) and N-251 (right panel) for 72 hrs, followed by real-time LightCycler PCR (black circles in the upper panel) and WST-1 assay (open triangles in the upper panel). The relative value (%) calculated at each point, when the level in non-treated cells was assigned as 100%, is presented here. Data are expressed as the means±standard deviation of triplicate assays. Western blot analysis (lower panels) was performed as described in Fig. 1B. (C) N-89 did not inhibit the HCV-JFH-1 replication. RSc (left panel) and D7 (right panel) cells were inoculated with supernatant from RSc cells replicating JR/C5B/BX-2 [29]. The RL assay was performed as described in Fig. 1B. (D) N-89 (left panel) and N-251 (right panel) did not inhibit the replication of HCV-JFH-1 subgenomic replicon. The RL and WST-1 assays were performed as described in Fig. 1B.

The genome-length HCV-RNA used in the assay systems described above contains three non-natural elements: RL, neomycin phosphotransferase, and an internal ribosomal entry site of encephalomyocarditis virus. To exclude the possibility that the anti-HCV activity of N-89 or N-251 was due to the inhibition of these three exogenous elements, we examined the anti-HCV activities of N-89 and N-251 using the authentic 9.6 kb HCV-RNA-replicating HCV-O/RLGE cells [19], which were developed by the introduction of *in vitro* synthesized HCV-O/RLGE RNA (Fig. 2A) into OR6c cured cells. We could demonstrate by quantitative RT-PCR and Western blot analyses that N-89 and N-251 at the expected concentrations efficiently prevented HCV-RNA replication and HCV Core expression in HCV-O/RLGE cells in a dose-dependent manner, respectively (Fig. 2B). The EC_50_ and SI values of N-89 and N-251 in this assay were calculated as follows each: EC_50_ 2.0 µM and SI >5.0 in N-89; EC_50_ 1.6 µM and SI 2.8 in N-251. To further confirm that N-89 or N-251 does not inhibit the RL activity, we examined the direct effect of each reagent by adding it along with substrate to the cell lysate in the RL assay. No suppressive effects by N-89 and N-251 were observed in either the OR6 assay (Fig. S2A) or the ORL8 assay (Fig. S2B). These results indicate that the anti-HCV activities of N-89 and N-251 were due to the inhibition of HCV-RNA itself, but not to exogenous elements contained in the genome-length HCV-RNA.

### N-89 and N-251 did not Inhibit RNA Replication of HCV-JFH-1 Strain

We next examined whether N-89 and N-251 worked in an HCV production system using HCV-JFH-1 strain (genotype 2a). Unexpectedly, the results using the JFH-1 reporter assay systems [29], which were recently developed using HuH-7-derived RSc and Li23-derived D7 cells, revealed that both N-89 and N-251 did not show anti-HCV activity for the HCV-JFH-1 strain (Fig. 2C, Fig. S3). To clarify whether anti-HCV activity depends on the difference of genotype or assay model, we evaluated the activities of N-89 and N-251 using RSc-JRN/35B [Ikeda et al., submitted] cells harboring a subgenomic HCV-JFH-1 replicon as an additional assay. The results revealed that N-89 and N-251 did not show any anti-HCV activities in this assay system either (Fig. 2D). Although the relative value of WST-1 almost became zero when RSc-JRN/35B cells were treated with 10 µM of N-251, cell counting after trypan blue dye treatment revealed that approximately 30% of the cells were viable (data not shown). These results suggest that the inhibitory effect of N-89 or N-251 on HCV-RNA replication may depend on genotype 1b or not work for only JFH-1 strain.

### OR6 and ORL8 Cells were Cured by Treatment with only N-89

**Figure 3:**
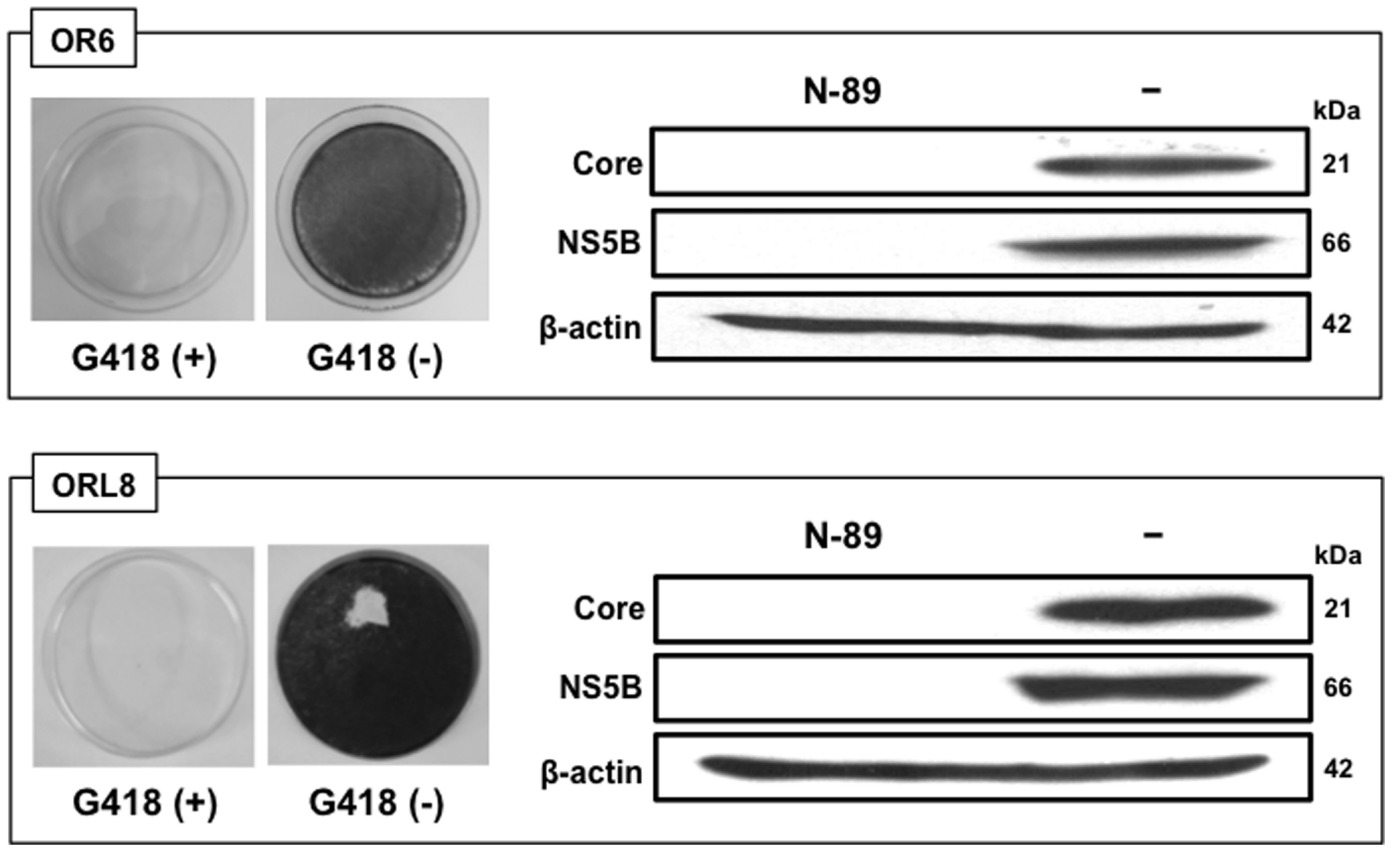
OR6 and ORL8 cells were cured by treatment with only N-89. The treated cells were divided into two plates with or without G418, and then cultured for 2 weeks. The left panels show the cells stained with Coomassie brilliant blue. The right panels show the results of Western blot analysis of the treated and non-treated cells for HCV proteins. Western blot analysis was performed as described in Fig. 1B.

To date, IFN-α alone or IFN-γ alone has generally been used to prepare cured cells from the cells harboring HCV-RNA [36]. Since we observed strong anti-HCV activity (>99% suppression) at 8 µM of N-89 in OR6 cells or 1 µM of N-89 in ORL8 cells without a decrease in cell viability (Fig. 1B), we expected that these cells might be cured only by treatment with N-89. Accordingly, OR6 and ORL8 cells were treated with 8 µM and 1 µM of N-89, respectively, in the absence of G418. The treatment was continued for 3 weeks with the addition of N-89 at 4-day intervals. All of the treated cells were dead when cultured in the presence of G418 for an additional two weeks, whereas the treated cells proliferated efficiently in the absence of G418 (Fig. 3), suggesting that OR6 and ORL8 cells are cured by monotherapy with N-89. This suggestion was confirmed by Western blot analysis (Fig. 3). These results indicate that N-89 is a strong anti-HCV reagent, which can be used to prepare cured cells by treatment at low concentration.

### Comparative Time Course Assay of the Anti-HCV Activities of N-89 and IFN-α

**Figure 4:**
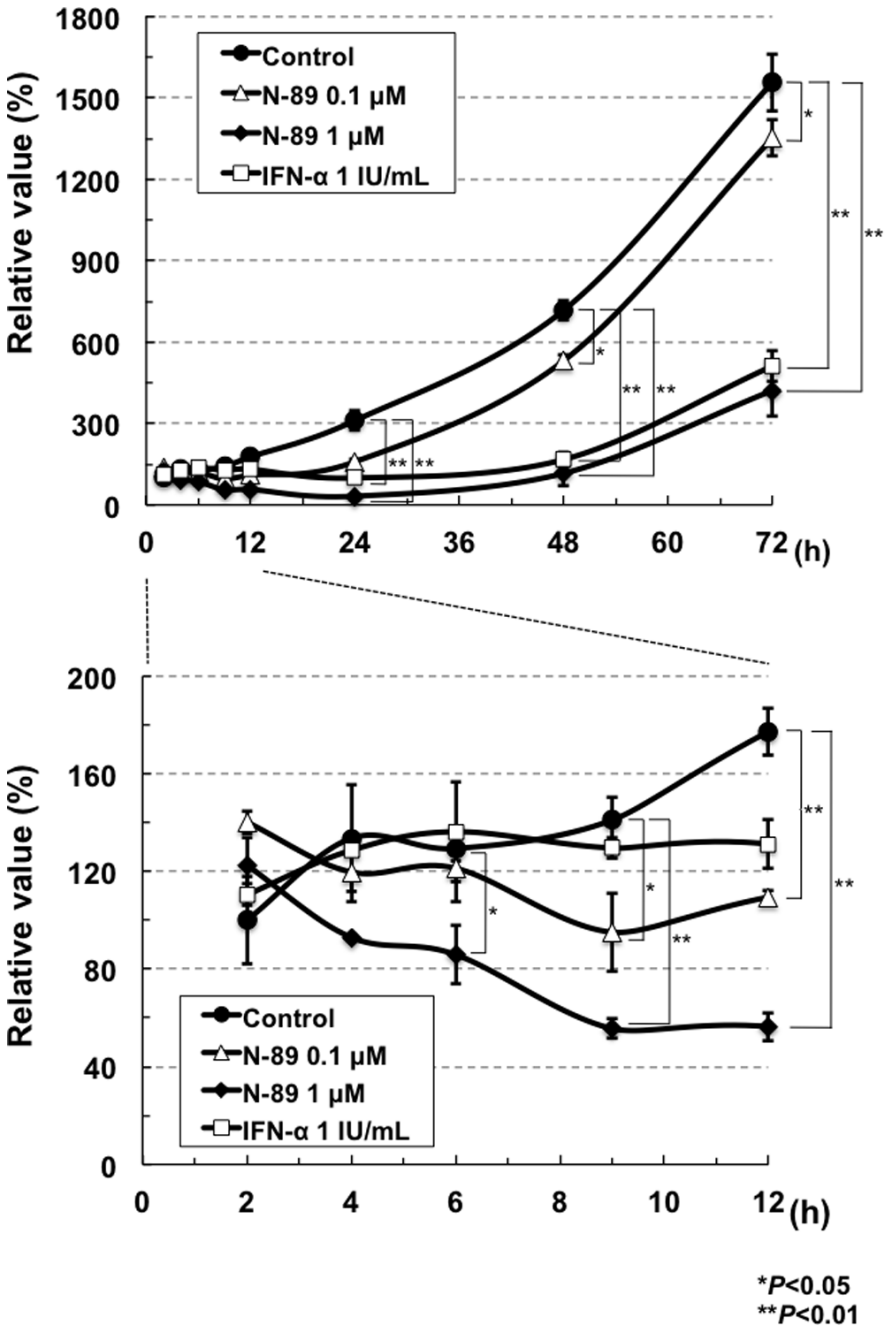
The anti-HCV action of N-89 was faster than that of IFN-α. The ORL8 cells were treated with N-89 or IFN-α, and then RL assays were performed at 2 to 72 hrs after the treatment. The relative value (%) calculated at each time point, when the luciferase activity of non-treated cells at 24 hrs was assigned as 100%, is shown. Data are expressed as the means±standard deviation of triplicate assays. The data within 12 hrs after the treatment are shown in the lower panel. *P<0.05; **P<0.01.**The** Anti**-HCV** Activities **of N-89 and N-251 were** Completely Canceled **by VE**We previously reported that the antioxidant VE canceled the anti-HCV activities of CsA and three nutrients (β-carotene, vitamin D_2_, and linoleic acid) [37], and demonstrated that the oxidative stress induced by these anti-HCV reagents caused anti-HCV status via activation of the extracellular signal-regulated kinase signaling pathway [38]. To evaluate this possibility, we examined the effect of VE on N-89 at the EC_90_ level in the ORL8 assay. CsA and IFN-α were also used as a positive and a negative control, respectively, on the effect of VE in the ORL8 assay. The results revealed that the anti-HCV activities of N-89 and CsA were largely canceled by VE, whereas the activity of IFN-α was not canceled (Fig. 5A). We normalized these results by dividing the RL value obtained in the presence of VE by that in the absence of VE as described previously [22], [37]. The values of N-89 and CsA were 16 and 34, respectively, whereas the value (3.2) of IFN-α was almost the same as that (3.0) of the control (Fig. 5B). Similar results were obtained by using N-251 (Fig. 5C and D). The values of N-251, CsA, and IFN-α were 13, 19, and 4.3, respectively, in comparison with the value (2.3) of the control (Fig. 5D). These results suggest that the induction of oxidative stress is associated with the anti-HCV activity of N-89 or N-251. However, an antimalarial drug, artemisinin, was hardly influenced by co-treatment with VE (Fig. 5E). The value (1.9) of artemisinin was almost the same as that (3.5 or 2.5) of IFN-α or the control, respectively (Fig. 5F). These results were also confirmed by Western blot analysis of HCV Core (Fig. 5G). Therefore, our results suggest that the anti-HCV mechanism of artemisinin is not associated with the induction of oxidative stress, and is distinct from that of N-89 or N-251.

We next performed a time course assay (2 to 72 hrs after treatment) in the case of ORL8 cells treated with N-89 (0.1 µM or 1 µM) or IFN-α (1 IU/ml; corresponding to approximately EC_80_). ORL8 cells treated with IFN-α (1 IU/ml) and N-89 (1 µM) had almost the same anti-HCV kinetics over the first 24 hrs after treatment (upper panel of Fig. 4); however, within the first 12 hrs after treatment N-89-treated ORL8 cells had more rapid anti-HCV kinetics than did the IFN-α-treated cells (lower panel of Fig. 4). N-89 at concentrations of 0.1 µM and 1 µM led to significantly decreased RL activity at 9 hrs and 6 hrs, respectively, after treatment, whereas a decrease of RL activity in the cells treated with 1 IU/ml of IFN-α began to be seen at 12 hrs after treatment (lower panel of Fig. 4). These results suggest that the action of N-89, and probably also that of N-251, is faster than that of IFN-α, and the anti-HCV mechanism of N-89 is different from that of IFN-α.

### Synergistic Effect of Anti-HCV Activity by N-89 or N-251 in Combination with IFN-α and/or RBV

**Figure 6:**
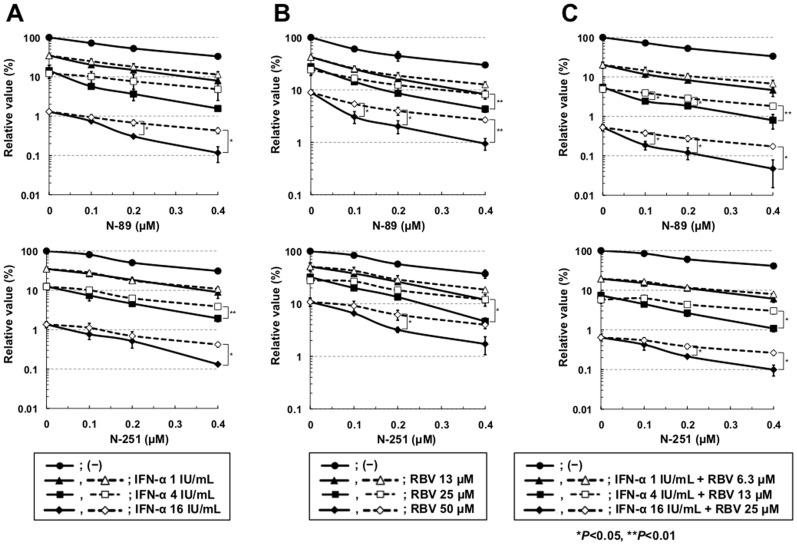
Synergistic anti-HCV effects of N-89 or N-251 in combination with IFN-α and/or RBV on HCV-RNA replication in ORL8 cells. Open symbols in the broken lines show the values expected as an additive anti-HCV effect and closed symbols in the solid lines show the values obtained by the ORL8 assay. ORL8 cells were treated with N-89 (upper panel) or N-251 (lower panel) in combination with IFN-α (A), RBV (B), or IFN-α and RBV (C) for 72 hrs and subjected to RL assay.

We examined the anti-HCV activity of N-89 or N-251 in combination with IFN-α using OR6 and ORL8 assay systems. The results of the ORL8 assay revealed that the anti-HCV activity of N-89 or N-251 in combination with IFN-α (more than 4 IU/ml) was significantly stronger than that expected as an additive effect, suggesting a synergistic effect of N-89 or N-251 and IFN-α (Fig. 6A). However, such an effect was not clear in the OR6 assay (Fig. S4A). We recently demonstrated that 10 µM (a clinically achievable concentration) of RBV efficiently inhibited HCV-RNA replication in the ORL8 assay [22], and demonstrated that adenosine kinase, which phosphorylates RBV to generate mono-phosphorylated RBV possessing the inhibitory activity for inosine monophosphate dehydrogenase, is an essential determinant of the anti-HCV activity of RBV in cell culture [23]. Therefore, we next examined the combination effect of RBV in the same way as IFN-α using an ORL8 assay. We observed that the anti-HCV activity of N-89 or N-251 in combination with RBV was significantly stronger than that expected additively, suggesting that there was a synergistic effect between N-89 or N-251 and RBV (Fig. 6B). However, in the OR6 assay, we noticed that RBV showed an additive anti-HCV effect in combination with N-89 or N-251 (Fig. S4B). Since RBV has been shown to have little anti-HCV activity in the OR6 assay system [22], some specific factor(s) in ORL8 cells might contribute to the synergistic effect of N-89 or N-251 in combination with RBV. Therefore, we further examined the effect of N89 or N-251 in combination with both IFN-α and RBV using an ORL8 assay. As expected, the anti-HCV activity of N-89 or N-251 was synergistically enhanced in combination with both IFN-α and RBV in the ORL8 assay (Fig. 6C). On the other hand, in the OR6 assay, a synergistic effect like that seen in the ORL8 assay was not observed (Fig. S4C). We confirmed that any such synergistic effect was not due to the cell toxic effect (Fig. S5).

## Discussion

N-89 and its derivative N-251 are preclinical and promising drugs possessing antimalarial activities *in vitro* and *in vivo* comparable to those of artemisinin [26], [27]. In the present study, using cell-based HCV-RNA-replication assay systems, we found that N-89 and N-251 possessed potent anti-HCV activities irrespective of the cell lines and HCV strains of genotype 1b, and that they did not work for JFH-1 strain of genotype 2a. Furthermore, We demonstrated that the anti-HCV kinetics of N-89 was faster than that of IFN-α, and that both N-89 and N-251 exhibited synergistic effects in combination with IFN-α and/or RBV.

Along with the worldwide spread of HCV, high prevalence areas of HCV infection have overlapped with endemic areas of malaria infection [39], [40]. It is also interesting that the liver is a target organ for the replication of HCV and malaria. This fact would again suggest that N-89 and N-251 target a common factor that is required for the replication of HCV and malaria. At the same time, N-89 and N-251 have become readily and cheaply available due to their ease of synthesis [26], [27]. Since we showed that HCV-RNA-replicating cells were cured by monotherapy with N-89, monotherapy with N-89 or N-251 would be simultaneously effective for the diseases caused by malaria and HCV infection. Furthermore, we recently showed that the blood concentration of N-89 or N-251 reaches approximately 1 µM [Kim et al., unpublished data]. Since this concentration, which is equivalent to the EC_99_ value of N-89 in the ORL8 assay, was used for the preparation of cured cells, even monotherapy with N-89 would be useful for patients with chronic hepatitis C.

In regard to the anti-HCV mechanism of N-89 and N-251, we provided evidence that the anti-HCV activity of these reagents was canceled by antioxidant VE, suggesting the induction of oxidative stress. To identify the target factor(s) located downstream of ROS production, we attempted microarray analysis using OR6 and ORL8 cells treated with N-89. However, consequently, we failed to obtain the candidate gene indicating the meaningful expression level, although we identified several genes, which were commonly upregulated or downregulated in the N-89-treated cells (Fig. S6). On the other hand, it has been recently reported that *Plasmodium falciparum* endoplasmic reticulum-resident calcium binding protein is a possible target of N-89 and N-251 [41]. Therefore, this protein may be involved in the anti-HCV activities of N-89 and N-251. To clarify the factor(s), further analysis will be needed.

The synergistic anti-HCV effect of N-89 or N-251 in combination with RBV rather than IFN-α is also interesting. Using RBV-sensitive ORL8 cells, we recently clarified that the anti-HCV mechanism of RBV was mediated by the inhibition of IMPDH, which is required for HCV-RNA replication [22]. In addition, since RBV is an important component of current IFN-based therapies, including the recently developed triple therapy, the use of N-89 or N-251 may further enhance the SVR rate achieved with the current therapy. Furthermore, recent report [42] that the *lead-in* four weeks of RBV treatment before starting a standard course of PEG-IFN with RBV led a weak decrease of viral replication (0.5±0.5 log_10_) is noteworthy. To evaluate this possibility, we compared the SI values of N-89, N-251, RBV, and CsA using the ORL8 assay system. The results revealed that the SI values of N-89, N-251, RBV, and CsA were 26, 13, 10, and 15, respectively, indicating that the anti-HCV activity of N-89 or N-251 is equivalent to that of RBV or CsA. Since the treatment with N-89/N-251 and RBV exhibits a synergistic effect, oral N-89 or N-251 would be good compounds for inclusion in the current triple therapy.

In conclusion, we found that two oral antimalarial drugs in the preclinical stage of development (N-89 and N-251) exhibited strong anti-HCV activities to genotype 1b. These compounds would have potential as one component of a therapeutic regimen based on combinations of HCV-specific inhibitors.

## Supporting Information

Figure S1 **Anti-HCV activities of N-89 and N-251 detected in the several assay systems using genome-length HCV-RNA or HCV subgenomic replicon RNA.** (A) Effects of N-89 and N-251 on genome-length HCV-RNA (AH1 strain of genotype 1b) replication in the AH1R assay. AH1R cells were treated with N-89 or N-251 for 72 hrs, followed by RL assay (black circles) and WST-1 assay (open triangles). The relative value (%) calculated at each point, when the level in non-treated cells was assigned as 100%, is presented here. Data are expressed as the means ± standard deviation of triplicate assays. (B) Effects of N-89 and N-251 on genome-length HCV-RNA (HCV 1B-4 strain of genotype 1b) replication in the 1B-4R assay. The RL assay and WST-1 assay were performed as described in (A). (C) Effects of N-89 and N-251 on genome-length HCV-RNA (HCV 1B-4 strain of genotype 1b) replication in the 1B-4RL assay. The RL assay and WST-1 assay were performed as described in (A). (D) Effects of N-89 and N-251 on genome-length HCV-RNA (HCV KAH5 strain of genotype 1b) replication in the KAH5RL assay. The RL assay and WST-1 assay were performed as described in (A). (E) Effects of N-89 and N-251 on HCV subgenomic replicon RNA (HCV O strain of genotype) replication in the sOR assay. The RL assay and WST-1 assay were performed as described in (A). (F) Effects of N-89 and N-251 on HCV subgenomic replicon RNA (HCV O strain of genotype 1b) replication in the sORL8 assay. The RL assay and WST-1 assay were performed as described in (A). (G) Effects of N-89 and N-251 on HCV subgenomic replicon RNA (HCV O strain of genotype 1b) replication in the sORL11 assay. The RL assay and WST-1 assay were performed as described in (A).
(TIF)

Figure S2 **No inhibition of RL activity by N-89 or N-251.** (A) N-89 and N-251 did not inhibit the RL activity in the OR6 cell lysate. N-89 or N-251 was added to the OR6 cell lysate, and then an RL assay was performed. (B) N-89 and N-251 did not inhibit the RL activity in the ORL8 cell lysate. N-89 or N-251 was added to the ORL8 cell lysate, and then an RL assay was performed.
(TIF)

Figure S3 **N-251 did not inhibit the HCV-JFH-1 replication.** RSc and D7 cells were inoculated with supernatant from RSc cells replicating JR/C5B/BX-2 [2]. The RL assay was performed as described in Fig. S1A.
(TIF)

Figure S4 **Anti-HCV effects of N-89 or N-251 in combination with IFN-α and/or RBV on HCV-RNA replication in OR6 cells.** Open symbols in the broken lines show the values expected as an additive anti-HCV effect and closed symbols in the solid lines show the values obtained by the OR6 assay. (A) Effect of N-89 or N-251 in combination with IFN-α on OR6 assay. OR6 cells were treated with N-89 (upper panel) or N-251 (lower panel) in combination with IFN-α for 72 hrs and subjected to RL assay. (B) Effect of N-89 or N-251 in combination with RBV on OR6 assay. OR6 cells were treated with N-89 (upper panel) or N-251 (lower panel) in combination with RBV for 72 hrs and subjected to RL assay. (C) Effect of N-89 or N-251 in combination with IFN-α and RBV on OR6 assay. OR6 cells were treated with N-89 (upper panel) or N-251 (lower panel) in combination with IFN-α and RBV for 72 hrs and subjected to RL assay.
(TIF)

Figure S5 **Effects of N-89 or N-251 in combination with IFN-α and/or RBV on the growth of ORL8 or OR6 cells.** ORL8 cells (A, B) or OR6 cells (C, D) were treated with N-89 (A, C) or N-251 (B, D) in combination with IFN-α for 72 hrs and subjected to the cell counting. The cell counting was carried out as described in the Supporting Materials and methods.
(TIF)

Figure S6 **Selection of genes whose expression levels were commonly upregulated or downregulated in the N-89-treated OR6 and ORL8 cells.** (A) Genes whose expression levels were upregulated at ratios of more than 2 in the case of OR6(−) versus OR6(N-89) or ORL8(−) versus ORL8(N-89) were selected. 4 genes upregulated commonly in the N-89-treated cells were listed. (B) Genes whose expression levels were downregulated at ratios of less than 0.5 in the case of OR6(−) versus OR6(N-89) or ORL8(−) versus ORL8(N-89) were selected. 5 genes downregulated commonly in the N-89-treated cells were listed.
(TIF)

Text S1. (DOC)
