# Correction: New Preclinical Antimalarial Drugs Potently Inhibit Hepatitis C Virus Genotype 1b RNA Replication

**DOI:** 10.1371/annotation/6e1f1f8f-c421-4def-aaf1-4ce7f4915d87

**Published:** 2013-09-27

**Authors:** Youki Ueda, Midori Takeda, Kyoko Mori, Hiromichi Dansako, Takaji Wakita, Hye-Sook Kim, Akira Sato, Yusuke Wataya, Masanori Ikeda, Nobuyuki Kato

As a result of errors in the typesetting process, a subsection from the "Results" section of the article was moved into the Figure 4 legend. The subsection comes after "Comparative Time Course Assay of the Anti-HCV Activities of N-89 and IFN-α" and is available in full below.

**The anti-HCV activities of N-89 and N-251 were completely canceled by VE**

 We previously reported that the antioxidant VE canceled the anti-HCV activities of CsA and three nutrients (β-carotene, vitamin D2, and linoleic acid) [37], and demonstrated that the oxidative stress induced by these anti-HCV reagents caused anti-HCV status via activation of the extracellular signal-regulated kinase signaling pathway [38]. To evaluate this possibility, we examined the effect of VE on N-89 at the EC90 level in the ORL8 assay. CsA and IFN-α were also used as a positive and a negative control, respectively, on the effect of VE in the ORL8 assay. The results revealed that the anti-HCV activities of N-89 and CsA were largely canceled by VE, whereas the activity of IFN-α was not canceled (Fig. 5A). We normalized these results by dividing the RL value obtained in the presence of VE by that in the absence of VE as described previously [22, 37]. The values of N-89 and CsA were 16 and 34, respectively, whereas the value (3.2) of IFN-α was almost the same as that (3.0) of the control (Fig. 5B). Similar results were obtained by using N-251 (Fig. 5C and D). The values of N-251, CsA, and IFN-α were 13, 19, and 4.3, respectively, in comparison with the value (2.3) of the control (Fig. 5D). These results suggest that the induction of oxidative stress is associated with the anti-HCV activity of N-89 or N-251. However, an antimalarial drug, artemisinin, was hardly influenced by co-treatment with VE (Fig. 5E). The value (1.9) of artemisinin was almost the same as that (3.5 or 2.5) of IFN-α or the control, respectively (Fig. 5F). These results were also confirmed by Western blot analysis of HCV Core (Fig. 5G). Therefore, our results suggest that the anti-HCV mechanism of artemisinin is not associated with the induction of oxidative stress, and is distinct from that of N-89 or N-251.

