# Supplementary figures and images for: New Preclinical Antimalarial Drugs Potently Inhibit Hepatitis C Virus Genotype 1b RNA Replication
Source: PLoS One. 2013 Aug 30;8(8):e72519. doi: 10.1371/journal.pone.0072519 (PMC3758303; doi:10.1371/journal.pone.0072519)

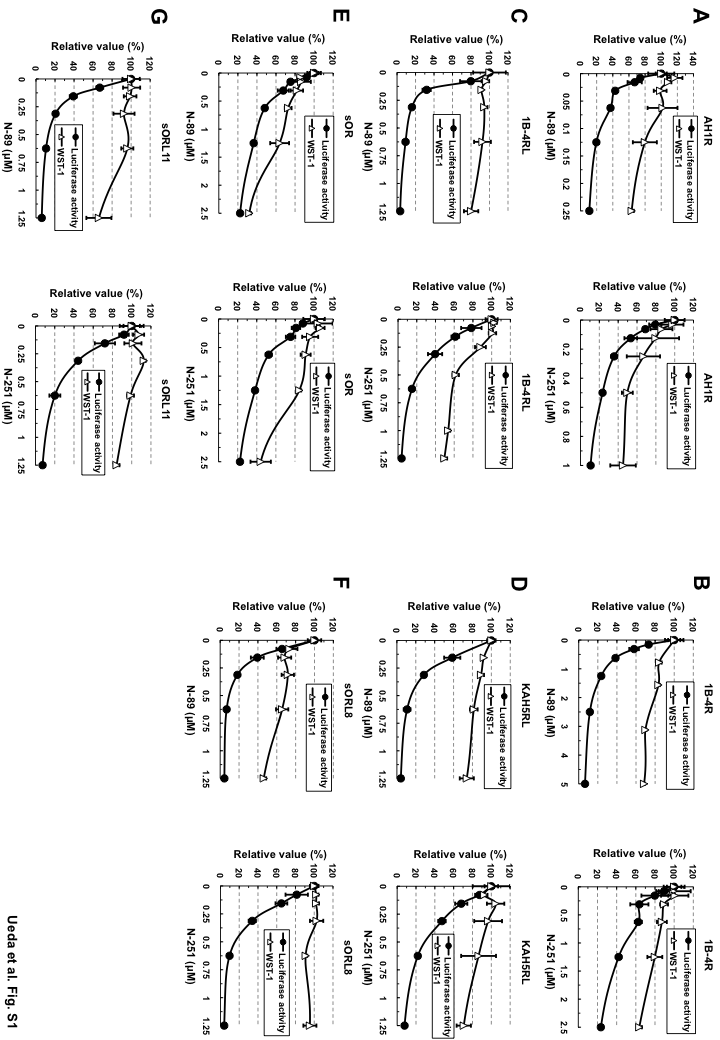

Supplement: Figure S1 — Anti-HCV activities of N-89 and N-251 detected in the several assay systems using genome-length HCV-RNA or HCV subgenomic replicon RNA. (A) Effects of N-89 and N-251 on genome-length HCV-RNA (AH1 strain of genotype 1b) replication in the AH1R assay. AH1R cells were treated with N-89 or N-251 for 72 hrs, followed by RL assay (black circles) and WST-1 assay (open triangles). The relative value (%) calculated at each point, when the level in non-treated cells was assigned as 100%, is presented here. Data are expressed as the means ± standard deviation of triplicate assays. (B) Effects of N-89 and N-251 on genome-length HCV-RNA (HCV 1B-4 strain of genotype 1b) replication in the 1B-4R assay. The RL assay and WST-1 assay were performed as described in (A). (C) Effects of N-89 and N-251 on genome-length HCV-RNA (HCV 1B-4 strain of genotype 1b) replication in the 1B-4RL assay. The RL assay and WST-1 assay were performed as described in (A). (D) Effects of N-89 and N-251 on genome-length HCV-RNA (HCV KAH5 strain of genotype 1b) replication in the KAH5RL assay. The RL assay and WST-1 assay were performed as described in (A). (E) Effects of N-89 and N-251 on HCV subgenomic replicon RNA (HCV O strain of genotype) replication in the sOR assay. The RL assay and WST-1 assay were performed as described in (A). (F) Effects of N-89 and N-251 on HCV subgenomic replicon RNA (HCV O strain of genotype 1b) replication in the sORL8 assay. The RL assay and WST-1 assay were performed as described in (A). (G) Effects of N-89 and N-251 on HCV subgenomic replicon RNA (HCV O strain of genotype 1b) replication in the sORL11 assay. The RL assay and WST-1 assay were performed as described in (A). (TIF) [file pone.0072519.s001.tif]

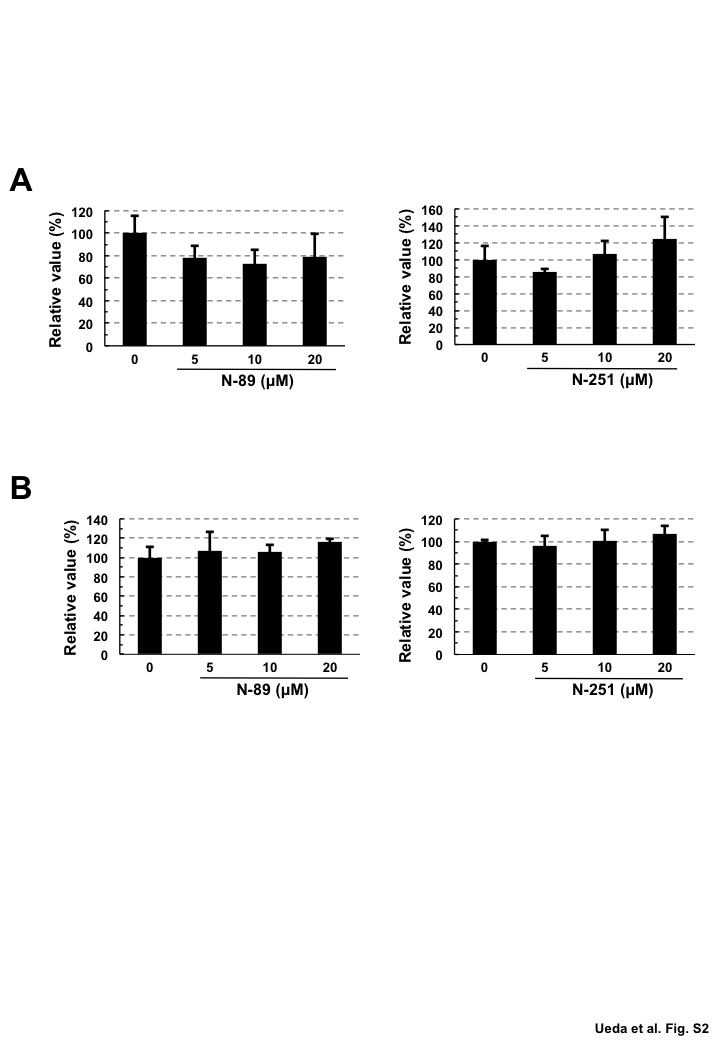

Supplement: Figure S2 — No inhibition of RL activity by N-89 or N-251. (A) N-89 and N-251 did not inhibit the RL activity in the OR6 cell lysate. N-89 or N-251 was added to the OR6 cell lysate, and then an RL assay was performed. (B) N-89 and N-251 did not inhibit the RL activity in the ORL8 cell lysate. N-89 or N-251 was added to the ORL8 cell lysate, and then an RL assay was performed. (TIF) [file pone.0072519.s002.tif]

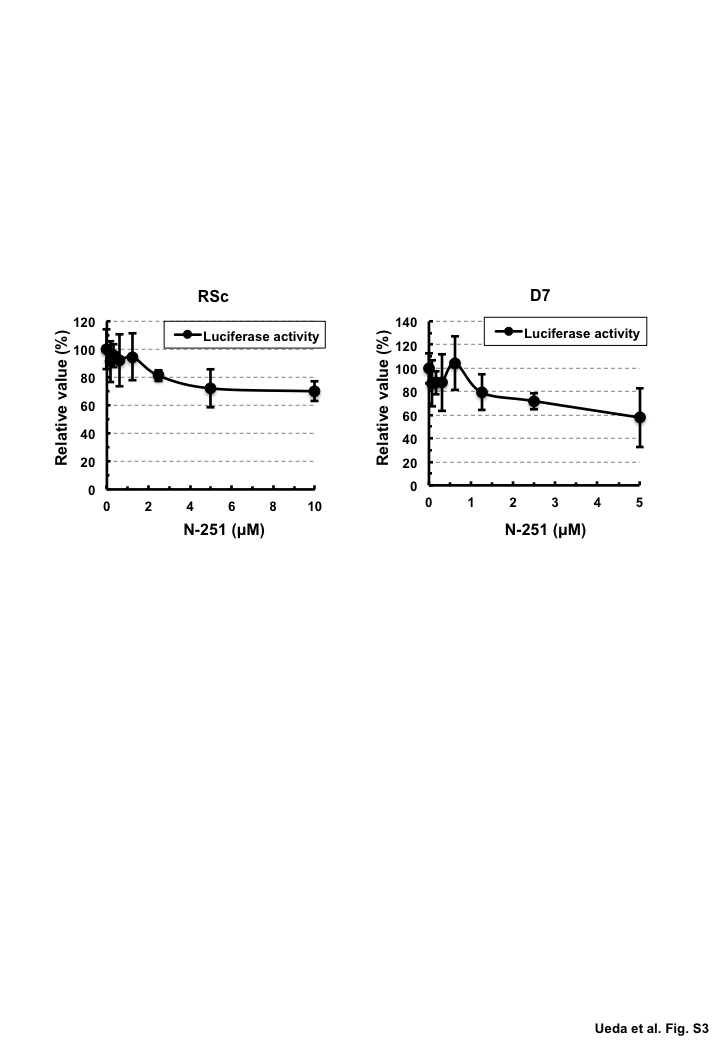

Supplement: Figure S3 — N-251 did not inhibit the HCV-JFH-1 replication. RSc and D7 cells were inoculated with supernatant from RSc cells replicating JR/C5B/BX-2 [2]. The RL assay was performed as described in Fig. S1A. (TIF) [file pone.0072519.s003.tif]

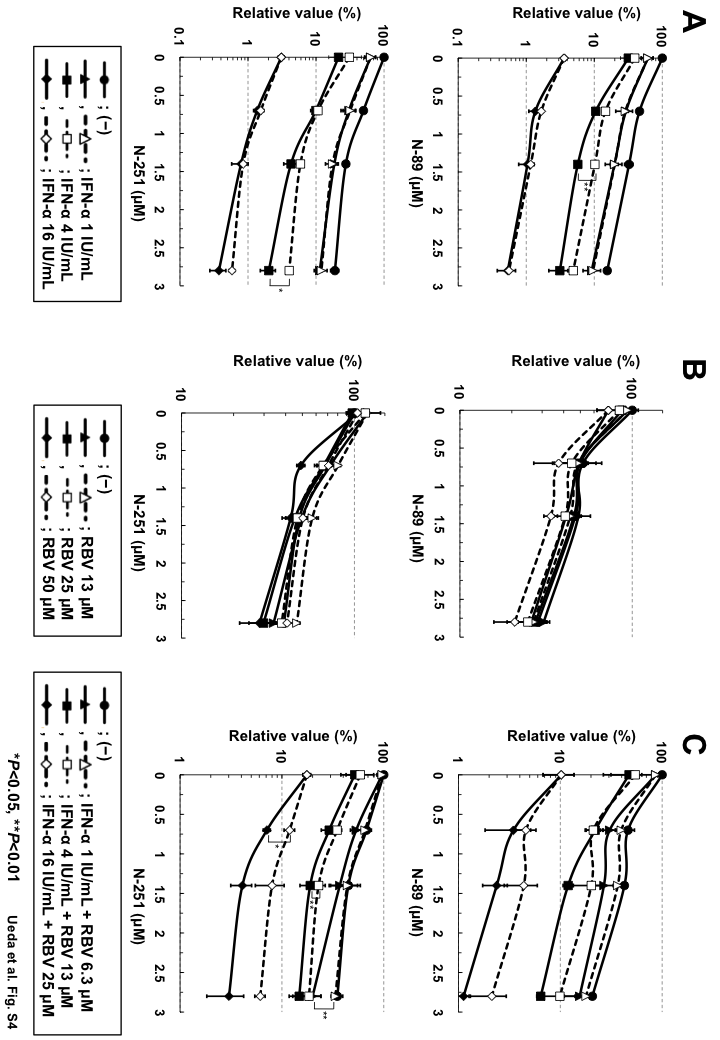

Supplement: Figure S4 — Anti-HCV effects of N-89 or N-251 in combination with IFN-α and/or RBV on HCV-RNA replication in OR6 cells. Open symbols in the broken lines show the values expected as an additive anti-HCV effect and closed symbols in the solid lines show the values obtained by the OR6 assay. (A) Effect of N-89 or N-251 in combination with IFN-α on OR6 assay. OR6 cells were treated with N-89 (upper panel) or N-251 (lower panel) in combination with IFN-α for 72 hrs and subjected to RL assay. (B) Effect of N-89 or N-251 in combination with RBV on OR6 assay. OR6 cells were treated with N-89 (upper panel) or N-251 (lower panel) in combination with RBV for 72 hrs and subjected to RL assay. (C) Effect of N-89 or N-251 in combination with IFN-α and RBV on OR6 assay. OR6 cells were treated with N-89 (upper panel) or N-251 (lower panel) in combination with IFN-α and RBV for 72 hrs and subjected to RL assay. (TIF) [file pone.0072519.s004.tif]

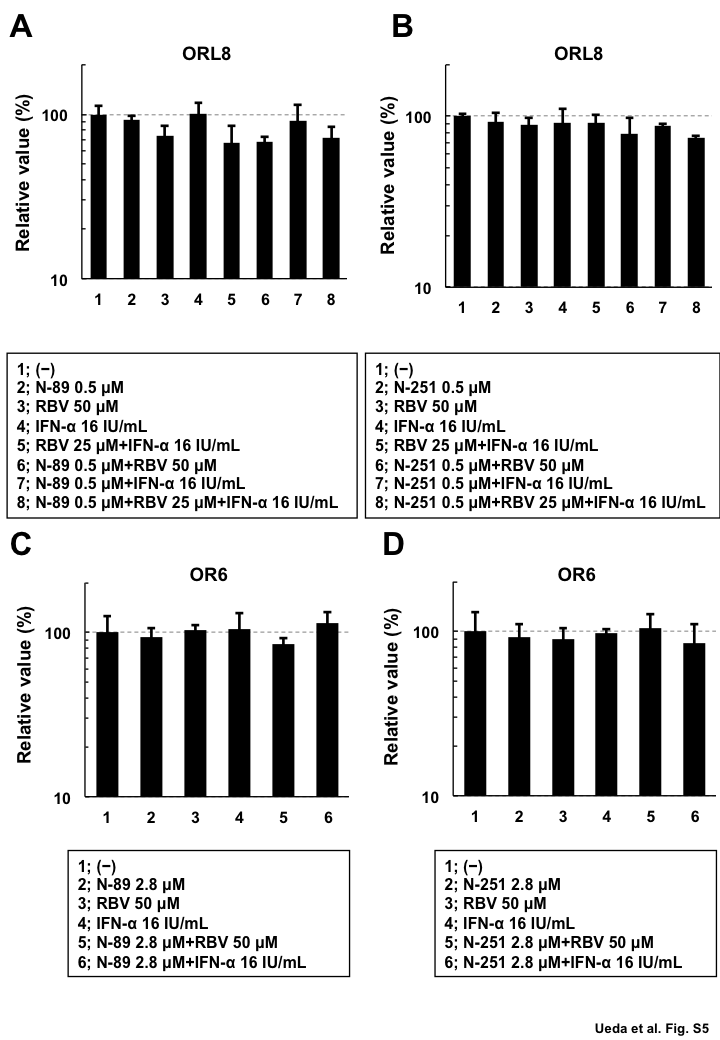

Supplement: Figure S5 — Effects of N-89 or N-251 in combination with IFN-α and/or RBV on the growth of ORL8 or OR6 cells. ORL8 cells (A, B) or OR6 cells (C, D) were treated with N-89 (A, C) or N-251 (B, D) in combination with IFN-α for 72 hrs and subjected to the cell counting. The cell counting was carried out as described in the Supporting Materials and methods. (TIF) [file pone.0072519.s005.tif]

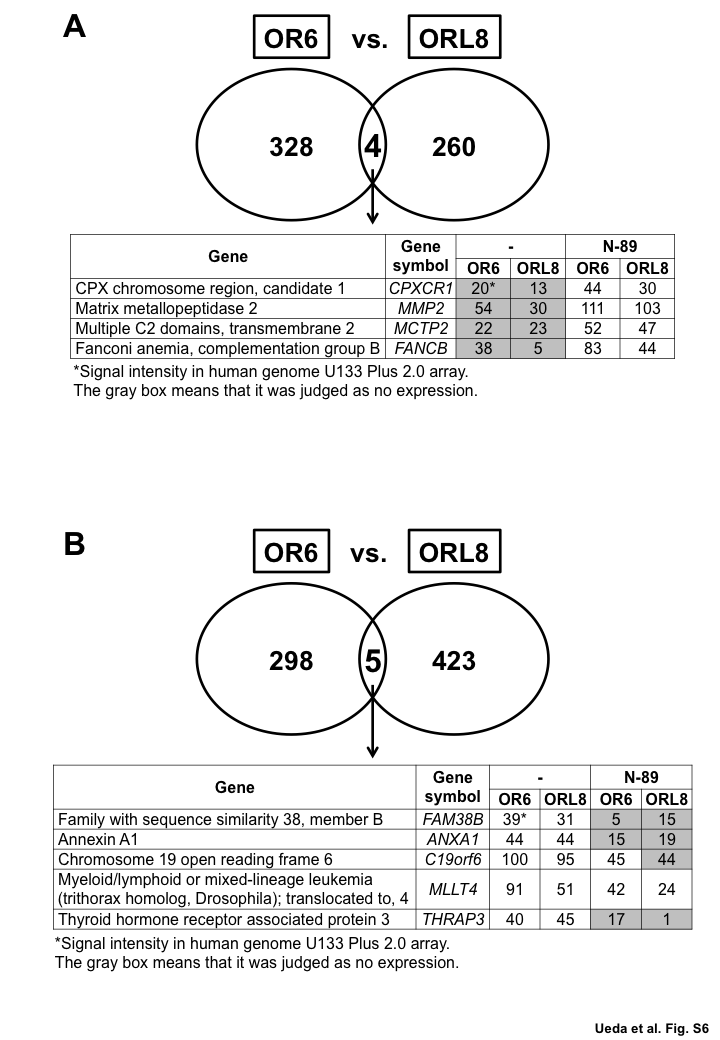

Supplement: Figure S6 — Selection of genes whose expression levels were commonly upregulated or downregulated in the N-89-treated OR6 and ORL8 cells. (A) Genes whose expression levels were upregulated at ratios of more than 2 in the case of OR6(−) versus OR6(N-89) or ORL8(−) versus ORL8(N-89) were selected. 4 genes upregulated commonly in the N-89-treated cells were listed. (B) Genes whose expression levels were downregulated at ratios of less than 0.5 in the case of OR6(−) versus OR6(N-89) or ORL8(−) versus ORL8(N-89) were selected. 5 genes downregulated commonly in the N-89-treated cells were listed. (TIF) [file pone.0072519.s006.tif]
