# Supplementary material for: New Preclinical Antimalarial Drugs Potently Inhibit Hepatitis C Virus Genotype 1b RNA Replication
Source: PLoS One. 2013 Aug 30;8(8):e72519. doi: 10.1371/journal.pone.0072519 (PMC3758303; doi:10.1371/journal.pone.0072519)
Supplement: Text S1. — (DOC) [file pone.0072519.s007.doc]

**Supporting Materials and Methods**

cDNA Microarray Analysis

OR6 and ORL8 cells (1 × 10^6^ each) were plated onto 10-cm diameter dishes (approximately 70-80% confluency), and then non-treated or treated with N-89 (5 μM for OR6; 1 μM for ORL8) for 6 hrs. Total RNAs from these cells were prepared using the RNeasy extraction kit (QIAGEN, Hilden, Germany). As previously described [1], cDNA microarray analysis was performed by Dragon Genomics Center of Takara Bio. (Otsu, Japan) through an authorized Affymetrix service provider using the GeneChip Human Genome U133 Plus 2.0 Array. Differentially expressed genes were selected by comparing the arrays from the non-treated and treated cells.

**Supporting References**

1. Kato N, Mori K, Abe K, Dansako H, Kuroki M, et al. (2009) Efficient replication systems for hepatitis C virus using a new human hepatoma cell line. Virus Res 146: 41-50.

2. Takeda M, Ikeda M, Ariumi Y, Wakita T, Kato N (2012) Development of hepatitis C virus production reporter-assay systems using two different hepatoma cell lines. J Gen Virol 93: 1422-1431.
